# Proceedings of Tenth Annual Session at Nashville

**Published:** 1857-06

**Authors:** 


### American Medical Association.

TENTH ANNUAL SESSION.

NASHVILLE, May 5, 1857.

The tenth annual session of the American Medical Association convened at the Representative's Hall in the State Capitol, at 11, A. M., yesterday.

Dr. ZINA PITCHER, of Michigan, assumed the chair as President, assisted by Vice President W. K. BOWLING, of this city.

Dr. Wm. Brodie, of Michigan, and Dr. R. C. Foster, of this city, Secretaries, were present.

The Chair stated the first business of the Association to be the reception of the report of the Committee of Arrangements, and Dr. C. K. Winston, of this city, as chairman of that committee, reported accordingly, and on behalf of his committee, and the medical profession of the city, extended a graceful and hearty welcome to the Association in a brief address.

On motion of Dr. C. K. Winston, the roll of delegates who had registered there names was read, from which it appeared that twenty States were represented by an aggregate of 146 delegates.

Upon the suggestion of Dr. C. K. Winston, our venerable fellow citizens, Drs. Felix Robertson, John Shelby, and John Overton were made permanent members of the Association.

The Convention then took a recess of fifteen minutes for the purpose of allowing the respective delegations to appoint each a member of the committee on nominations. Subsequently the nominating committee was reported and organized as follows:

Connecticut, Chas. Hooper; New Hampshire, A Smally; Indiana, W. W. Hitt; Wisconsin, J. K. Bartlett; New York, Jas. R. Wood; Michigan, A. B. Palmer; Missouri, J. S. Moore; Illinois, T. K. Edmiston; Kentucky, R. J. Breckinridge; Arkansas, T. G. McGawock; Ohio, B. S. Brown; S. Carolina, R. W. Gibbes; Alabama, W. P. Reese; Mississippi, F. B. Shuford; New Jersey, R. M. Cooper; Louisiana, S. O. Scruggs; Pennsylvania, P. Cassidy; Georgia, Thos. S. Powell; Tennessee, J. B. Lindsley.

The committee upon nominations having been organized, the President, Dr. Pitcher, delivered his annual address. The address was received with manifestations of pleasure and approbation.

The Hon. Judge Catron was invited to a seat within the bar of the Association.

The Chairman of the Committee of Arrangements announced that the sessions of the Association would be from 9 oclock A. M. to 2 P. M.

The nominating committee was allowed to retire for the purpose of presenting the names of officers of the Association.

On motion, the thanks of the Association were tendered to the President for his very able address, and the same was referred to the Committee on Publication.

The report of the Committee on Publication was then read.

The Committee on Prize Essays were allowed further time to report.

The President announced to the Convention that Drs. F. Stewart, Alden March, Isador Gluck, of New York, and Dr. Pancoast of Pennsylvania, had been appointed to represent this Association in foreign scientific bodies.

The Committee on Medical Education and Medical Literature had no report.

Dr. J. C. Watson, of Maine, read a communication on behalf of the committee on Medical Topography and Epidemics, asking further time which was granted.

Dr. Arnold of Georgia, offered a resolution constituting the committee on nominations a standing committee, to whom shall be referred all business not requiring immediate action. Adopted.

The Secretary read the apology of Dr. James Mauran, of the committee on Medical Topography and Epidemics, for Rhode Island, which was accepted.

Mr. Peregrine Worth, of same committee for Maryland, sent in his report, accompanied with reports from Drs. A. M. White, and Edmund E. Waters. Received and referred to committee on publication.

Dr. W. F. Sutton, of same committee, for Kentucky, apologized for the absence of a report.

The members of same committee were called from New Hampshire, Vermont, Massachusetts, New York, New Jersey, Pennsylvania, Delaware, Virginia, District of Columbia, South Carolina, North Carolina, Tennessee, the territory of Minnesota, neither of whom reported.

The Committee on Nominations reported through their chairman, Dr. J. B. Lindsley, the following officers for the ensuing year:

President—Dr. Paul F. Eve, of Tennessee.

Vice Presidents—R. J. Breckinridge, Ky.; W. H. Byford, Ind.; S. M. Reese, N. Y., and H. F. Campbell, Ga.

The report was concurred in.

The President announced that the Secretaries would be chosen when it was ascertained where the next meeting will be held.

On motion of Dr. Wistar, the Chair appointed a committee of three, consisting of Drs. Wistar, Arnold, and McGugin, to conduct the newly elected officers to their seats.

The President elect being absent, the Association adjourned to 9 A. M. to-day.

The following is a list of the delegates whose names were registered yesterday morning. Additional delegates are expected to-day.

Iowa.—Asa Horr, Wm. Watson, D. L. McGugin, J. C. Hughes.

SECOND DAY.

Nashville, May 6, 1857.

The Association met pursuant to adjournment. The minutes of yesterday were read and adopted.

The newly elected officers were then inducted to their respective seats.

Dr. Eve, of Tennessee, in taking the chair, addressed the Association in a few pertinent remarks.

Dr. Winston, of Tennessee, read the names of additional delegates to the Association.

Dr. Hooker, from the committee on Topography and Epidemics for the state of Connecticut, being called on for his report, arose and explained that it was his understanding that the committee were to have three years in which to make their report, and at the end of that time he would either be prepared or ask the indulgence of the Association for further time.

The President appointed Drs. Curry, Grant and Evans, a committee on voluntary contributions.

The report of Dr. Posey, of Georgia, on the same subject, being called for, Dr. Arnold, of Georgia, read an abstract of the report of Dr Posey; all of which, on motion of Dr. Palmer of Michigan, was referred to the committee on Publication, under a suspension of the rule.

On Motion of Dr. Wood, of New York, the reports which were presented yesterday were also referred to the committee on Publication, under a suspension of the rule.

The state of Ohio being called upon for a report upon its Medical Topography and Epidemics, the Secretary read an apology from Dr. G. Mendenhall, who asked further time in which to make a report, which was granted.

The states of Mississippi, Missouri, Michigan, Illinois, Indiana, Wisconsin, Iowa, California, and the U. S. Navy, being called, no response was made.

A telegraphic despatch from Dr. J. M. Semmes of New York, who was to report on the treatment of the results of Obstructed Labor, was received and referred to the appropriate committee.

A communication was received from the Southern Methodist Publishing House, inviting the members of the Association to visit that establishment, which was received and accepted.

A communication was read by Dr Lindsley, of Tennessee, from the Medical Society of Washington City, inviting the National Association to hold their next annual meeting in that city. On motion, the communication was referred to the committee on nominations.

A resolution was offered by Dr. Bartlett of Wisconsin, tendering a vote of thanks to the late President of the Association, Zina Pitcher, for the able manner in which he has presided over the deliberations of this body, which was unanimously adopted.

The reports of special committees for 1856–7, being next in order, they were called in order as follows :

Inflammation—its Pathology, etc.—Dr. E. R. Peaslee, of Maine, asked further time. Referred.

Anatomy and Histology of the Cervex Uteri.—Drs. H. Hutchinson and Charles E. Isaacs, New York ; no report.

Treatment of Cholera.—Dr. J. Taylor, Bradford, Ky. ; no report.

Treatment best adapted to each variety of Cataract, etc.—Dr. Mark Stephenson, New York ; further time asked. Referred.

Causes of the Impulse of the Heart, etc.—Dr. J. W. Carson, New York ; a communication received. Referred.

Causes of Infant Mortality, etc.—Dr. Meredith Roese, New York, read an abstract of his report, which was referred to the committee on Publication.

The venerable Dr. Shelby of Tennessee, being present, was invited to a seat on the stand. His appearance was warmly acknowledged.

Dr. Hobbs of Ill., offered the following resolution:

*Resolved,* That a committee on Essays (not including Prize Essay,) be appointed, to whom all essays prepared by members for publication by this Association shall be referred, which committee shall transfer to the committe on Publication, all essays they judge worth publishing. That said committee on essays make a full report of their proceedings at its next annual session; provided, authors being informed of said rejection, by said committee, shall have the privilege of withdrawing their essays from the report of the committee to the Association.

On motion of Dr. Palmer, of Michigan, the resolution was indefinitely postponed.

The Secretary read a protest signed by Drs. Richard Arnold, J. Gordon Harvard, Pike Brown, Geo P. Padford, against admitting the delegates from Oglethorpe Medical College. After explanations and some discussion,

On motion of Dr. Gunn of Michigan, the protest was laid upon the table, and on motion of Dr. Palmer, the whole subject was referred to a committee of three to be appointed by the Chair.

Dr. Brodie of Michigan, moved as an amendment, that no Faculty member of a medical college be appointed upon the committee, which was accepted by the mover.

The Chairman appointed as such committee, Drs. Wistar of Pa., Bemis of Ky., and Gibbs of South Carolina.

Dr. Felix Robertson, the oldest physician in Tennessee, being in attendance, was invited to a seat on the stand. He was greeted with marked consideration by the Association.

The committee on nominations was convened to transact important business.

The calling of special committees was resumed:

Spontaneous Umbilical Hemorrhage, etc.—Dr. J. Foster Jenkins, New York; further time asked. Referred.

Use of Instruments in Obstetrical Practice.—Dr. Henry Carpenter, Pennsylvania; no report.

Measures to be adopted to remedy the evils existing, in the present mode of holding Coronors' Inquests.—Dr. Alexander J. Semmes, D. C. Report presented and the following resolution offered:

*Resolved*, That committees of three in each state, territory, and the District of Columbia, be appointed, and that said committees be, and they are hereby authorized in the name of this Asssociation, to memorialize their respective legislatures, to pass such laws as will best carry into effect, the objects of the foregoing report.

The resolution was adopted and referred.

Treatment of the Results of Obstructed Labor.—Dr. J. Marion Sims, New York.—Previously acted upon.

True Position and Value of Operative Surgery, etc.—Dr. J. B. Flint, Kentucky. Further time asked ; granted.

Causes and Cure of Indigestion, etc.—Dr. G. Volney Dorsey, Ohio ; no report.

Medical Jurisprudence of Insanity etc.—Dr. C. B. Coventry, New York. Further time granted.

Human, Animal and Vegetable Parasites.—Dr. Joseph Leidy, Pennsylvania ; no report.

Value of Strict Attention to Position in the Treatment of Diseases of the Abdomen.—Dr. M. D. Darnall, Indiana ; no report.

Milk Sickness.—Dr. George Sutton, Indiana ; no report.

Blending and Conversion of the Types of Fever.—Dr. Clark G. Pease, Wisconsin. Communication sent, but not received. Postponed.

Best Substitutes of Cinchona, etc.—Dr. B. S. Woodworth, Indiana ; no report.

Use of Cinchona in Malarious Diseases.—Dr Eranklin Hinkle, Pennsylvania. Report furnished ; referred to committee on Publication.

Nervous System in Febrile Disease.—Dr Henry F Campbell, Georgia. Verbal abstract of report given.

Laws Governing the Absorption and Deposit of Bone.—Dr John Neill, Pennsylvania ; no report.

Intimate Effects of Certain Toxicological Agents in the Animal Tissues and Fluids.—Dr John W Green, New York ; no report.

Medical Topography and Fauna of Washington Territory.—Dr George Suckley, U S Army. Report presented and referred.

Flora of Washington and Oregon Territories.—Dr James Cooper, New Jersey. Report presented and referred.

Intimate Structure and Pathology of the Kidney.—Dr Charles E. Isaacs, New York ; further time granted.

Diseases Incident to Emigrants, etc.—Dr Israel Moses, New York ; no report.

Etiology and Pathology of Epidemic Cholera.—Dr T W Corbon, Ohio. Partial report presented and referred.

Excretions as an Index to the Changes going on in the System.—Dr J A Johnson, Illinois ; no report.

Remedial Effects of Choloroform.—Dr D D Thompson, Ky ; no report.

Best means of causing an Increase in the number of Essays, etc.—Drs Leidy, Wood and Meigs, Pa ; no report ; committee continued.

Changes Produced in Composition and Properties of Milk, etc.—Dr N S Davis, Illinois. Communication read and time granted.

Stomatitus Materna.—Dr McGugin, Iowa ; further time granted.

An extract of the report of Dr Fenner of La., upon the Medical Topography of that state was then read and referred.

Dr. Dungleton of Pennsylvania, offered the following resolution, which was unanimously adopted :

*Resolved,* That in the death of Dr Grafton, of Mississippi, the American Medical Association has lost a talented and useful member, and society a benefactor.

Dr Gaspar Wistar, chairman of the committee upon the admission of delegates from Oglethorpe Medical College, reported in favor of their admission, which report was adopted.

The Secretary read the following preamble and resolutions, which were unanimously adopted :

*Whereas,* It has pleased God to remove by death our fellow-member, Robert M Porter, and because of his devotion to the interests of the Profession of Medicine, and his steady support of the American Medical Association,

*Resolved,* That this Association learned with unfeigned sorrow of his decease ; and that they have lost a firm and intelligent supporter, and society a benefactor and a friend.

Dr T Bullard, of Indiana, offered the following :

*Resolved,* That in the death of Dr John L Mothersett, this Association has lost a talented and useful member, and society a benefactor.

The Secretary read a communication from the Connecticut Medical Society, asking that the time for holding the meetings of the Association in the northern cities, be changed to a later period in the year. Referred to the committee on nominations with instructions to make a report. Adjourned.

THIRD DAY.

NASHVILLE, May 7, 1857.

The Association met pursuant to adjournment.

The minutes of yesterday were read and adopted.

Dr Hoyt from the committee of Arrangements read the names of additional delegates to the Association, who had arrived since the meeting of the Association yesterday.

The Secretary read a communication from Dr Clark G Pease, of Wisconsin, which accompanied his report on "Blending and Conversion of the Types of Fever."

Dr Hooker of Connecticut, moved that the report be referred to the committee on voluntary contributions.

Dr McKinley moved to amend by having a portion of the report read, which was lost.

And the motion recurring to refer to the report, it was carried.

Dr Curry, from the committee on voluntary contributions, submitted the following report which was accepted :

The committee on voluntary contributions has examined the following papers and recommend them for publication in the Transactions of the Association :

1st. A new Principle of Diagnosis in dislocations of the shoulder joint : By L. A. Dugas, M. D., Professor of Surgery in the Medical College of Augusta, Ga., accompanied by four photographic plates, illustrating the principle.

2d. Medical statistics of Washington Territory : By Geo. Suckley, M D, U S A, embracing, 1st, Geological Divisions of the Territory; its Geology, Meteorology, Fauna. 2d, White population and its diseases. 3d, Native population, diseases, medical practice ; causes of its rapid disappearance. Concluding remarks.

3d. Medical Flora of Washington and Oregon Territories : By J G Cooper, M D.

All of which was respectfully submitted.

R O CURRX, Ch'n.
R T EVANE,
GEO R GRANY.

Dr Lindsley, from the nominating committee, submitted the following report :

Secretaries—Robert C Foster, Tenn ; A J Semmes, Washington City.

Treasurer—Gaspar Wistar, Philadelphia.

For the next place of meeting, Washington City.

STANDING COMMITTEES.

Committee on Publication—Francis G Smith, of Philadelphia, chairman; Gaspar Wistar, of Philadelphia; R C Foster, of Nashville; A J Semmes, Washington City; Samuel L Hollingsworth, of Philadelphia; Samuel Lewis, of Penn; H F Askew, of Delaware.

Committee on Prize Essays—Grafton Tyler, of Georgetown, D C, chairman; J C Hall, of D C; J F May, of D C; Thomas Miller, of D C; A J Semmes, of D C; W J C Duhamel, of D C.

Committee of Arrangements—Harvey Lindsley, chairman; W J C Duhamel, Cornelius Boyle, P H Coolridge, G M Dove, A Y P Garnett, Wm P Johnston, of D C.

Committee on Medical Education—G W Norris, of Philadelphia, chairman; A H Luce, of Ill; E. R. Henderson, of S C; G R Grant, of Tenn; T S Powell, of Ga.

Committee on Medical Literature—A B Palmer, of Detroit, chairman; A F Alexander, of Ala; J M Mosgrove, of Ohio; P Cassidy, of Penn; S Pollak, of Missouri.

Vacancies in Committee on Medical Topography and Epidemics—T B Shuford to fill the vacancy caused by the death of Dr Grafton, of Miss. C W Parsons to fill the vacancy caused by the resignation of Joseph Mauran, of Rhode Island.

SPECIAL COMMITTEES.

Spontaneous Umbilical Hemmorrhage of the Newly Born—J. Foster Jenkins, of New York.

Influence of Marriages of Consanguinity upon Offspring—Dr Bemis, of Ky.

Functions of different portions of the Cerebellum—E Andrews, of Illinois.

Causes of the impulse of the Heart and the agencies which influence it in health and disease—J W Corson, of New York city.

Treatment of the results of Obstructed Labor—J Marion Sims, of New York.

Treatment best adapted to each variety of Cataract, with the method of operation, place of election, time, age, etc.—Mark Stephenson, of New York.

Human, Animal, and Vegetable Parasites.—Dr. Joseph Leidy, of Piladelphia.

Best substitute for Cinchona and its preparations in the treatment of Intermittent Fever, etc.—B. S. Woodward, of Ind.

Intimate structure and Pathology of the Kidney.—Charles E. Isaacs, of N. Y.

Etiology and Pathology of Epidemic Cholera.—T. W. Gordon, of Georgetown, Broom Co., Ohio.

Inflammation of Cervix Uteri.—Henry H. Miller, of Louisville, Ky.

On Milk Sickness.—D. W. H. Byford.

Best means of causing an increased number of Essays.—Drs. Leidy, Wood, Meigs, of Pa.

Changes produced in composition and properties of Milk.—N. S. Davis, Ill.

Stomatitis Materna.—D. L. McGugin, of Iowa.

On criminal abortion, with a view to its general suppression.—H. N. Storer, of Boston.

The committee recommended that the committees ordered by the adoption of the resolution accompanying Dr. A. J. Semmes' report be filled by the several State Societies.

On motion of Dr. Brodie, amended so as to refer the same to the officers of the several State Societies. Carried.

The committee also recommend the amendment of the third article of the constitution, in relation to *meetings*, by inserting after the words "first Tuesday in May," the words, "or the first Tuesday in June," and also by inserting after the words, "shall be determined," the words "with the time of meeting."

Special committee on the present state of science, as regards the pathology and therupeutics of the reproductive organs of the female. —E. Fordyce Barker, of New York.

On Moral Insanity.—D. M. Reese, M. D., New York.

On Calculi and diseases of the Urinary Organs, in Iowa, Minnesota and Nebraska.—Dr. J. C. Hughes, of Keokuk.

On the nature, tendency, and general treatment of Syphilitic Bubo.—Dr. Gunn, of Detroit.

On Medical Education.—(By Dr. Curry's resolution,) James R. Wood, of New York; Geo. R. Grant, Memphis, Tenn; John Watson, New York; C. B. Nottingham, Macon, Ga; Rene la Roche, Philadelphia, Pa.

To fill a vacancy in committee on Medical Topography and Epidemics.—Dr. J. L. Cabell, Charlottsville, Va.

Dr. Marsh moved that the report of the nominating committee be taken up, and each subject to which it refers, be considered separately, which motion prevailed. That portion relating to nominations was then adopted.

The place of the next annual meeting of the Association being the next subject in order, after some discussion, on motion of Dr. Marsh, the report of the committee was adopted.

Dr. Lindsley moved that as Dr. Semmes, one of the newly elected Secretaries, was absent, Dr. Brodie, of Michigan, be elected Secretary, *pro tem.*, which was carried.

Dr. Pitcher offered the following resolution, which was unanimously adopted:

*Resolved,* That a committee of three be appointed, of which the President of the Association shall be the chairman, to communicate with the Surgeon General of the army, the chief of the Medical Bureau of the navy, and the Secretary of the Treasury of the United States, with a view to secure the concurrence of these departments of the Federal government, so that its contributions to the Medical Topography and Vital Statistics, and the Sanitary Police of the nation may be made tributary to the labors of this Association.

The chairman appointed as such committee, Drs. Zina Pitcher, of Michigan, and R. H. Coolidge, of Kansas.

Dr Yankell offered the following resolution:

*Resolved,* That this Association re-affirm the principles respecting the rights of constituent bodies announced in a report contained in Vol. V, of its Transactions in the following terms:

"The Faculty of every chartered Medical College shall have the privilege of sending two delegates to this Association, provided that the said Faculty contain not less than six Professors, who give one course of instruction annually, of not less than sixteen weeks, on Anatomy, Materia Medica, Theory and Practice of Surgery, Midwifery, and Chemistry, and also that the said Faculty requires that its candidates for graduation, among other requisites, shall have attended two full courses of lectures, with an interval of not less than six months between them, one of which courses must have been in their Institution.

Dr. Breckinridge in the chair,

Dr. Buchanan proceeded to discuss the resolution, and at the close of his remarks moved to lay it on the table, which was subsequently withdrawn.

Dr. Boring offered the following resolutions in lieu, which he proceeded to discuss:

*Resolved,* That this Association has not the power to control the subject of Medical Education.

*Resolved,* That the great objects of this Association are the advancement of Medical Science, and the promotion of harmony in the profession.

*Resolved,* That the attempt upon the part of this body, to regulate Medical Education, having most signally failed in its object, and already introduced elements of discord, any further interference with this subject would not only be useless, but calculated to disturb and distract the deliberations of the Association.

Dr. Curry offered the following resolutions in lieu of the whole subject:

*Whereas,* The subject of Medical Education has been committed at each annual session to standing committees, and various suggestions have been proposed which the Association has adopted, and recommended to private instructors and to the medical colleges,

*Resolved,* That a committee of five be appointed by the committee on nominations, as a Special Committee, to be composed of members who are in no respect connected with any medical school, to devise a system of Medical Instruction, to be presented for the consideration of this Association at its annual session in 1858.

*Resolved,* That the proposed system shall set forth a uniform basis upon which our medical institutions shall be organized, as well as have reference to the best method of securing the preparatory medical instruction to the student, and that consequently the legitimate subjects to be embraced in said system will include primary medical schools—the number of professorships in medical colleges, the length and number of terms during the year, the requisite qualifications for graduation, and such other subjects of a general character as to give uniformity to our medical system, and preserve harmony and friendly intercourse in the ranks of the profession.

*Resolved,* That, upon the adoption of the proposed system by the Association, all institutions which may conform to it shall be entitled to representation at the annual session of this Association and none others.

The subjuct was further discussed by several members of the Association.

Dr. Reese, after some remarks, moved the indefinite postponement of the whole subject, which was lost.

Dr. Arnold moved the previous question which was lost, and the discussion proceeded at considerable length, when

Dr. Hooker moved the previous question on the resolutions of Dr. Curry.

The reading of the various resolutions being called for, they were read to the Association.

The motion of Dr. Hooker being in order the previous question was called and the resolutions of Dr. Curry were adopted.

Dr. Bowling, Chairman of the Committee on Prize Essays, submitted the report of said Committee, as follows:

The Committee on Prize Essays report that four Essays have been received, each possessing great merit.

The Committee selected the following two Essays for the two prizes, provided for at the last meeting of this Association.

1st. One entitled "The Excreto-Secretory System of Nerves. Its relations to Physiology and Pathology," with the following motto:

"*Observation becomes Experiment when used in severe processes of Induction,*" and signed "Henry Fraser Campbell, Georgia."

2nd. "Experimental researches relative to the Nutrition, Value, and Physiological Effects of Albumen. Starch and gum when singly and exclusively used as food," with the following motto:

"*Qum sequimur? quone in jubes? ubi ponere sedis?*

*Da pater augurium, atque animis illabere nostris!*" and signed William A. Hummond, M. D., Assistant Surgeon U. S. Army.

The President read an invitation to the members of the Association to visit the University of Nashville, in its military, literary, and medical departments.

The Committee on Voluntary Contributions, reported in favor of the publication in the transactions of the Association, of the following paper:

"On the blending and conversion of Types in Fever. By C. S. Pease, M. D., of Wisconsin."

The report was adopted.

Dr. McMurray offered the following resolution which was adopted:

*Resolved*, By this Association, that the Committee on Publications be instructed to append the Code of Ethics of the American Medical

Association to each volume of its present and future Annual Transactions.

The amendments to the Constitution proposed by Dr. Stocker, of Pa., at the last annual session, were taken up and laid on the table.

Dr. Lindsley offered the following amendment to the Constitution, which was seconded by Dr. Gunn :

"In Art, II, omit the words 'Medical Colleges,' and also the words 'The Faculty of every Medical College, or Chartered School of Medicine, shall have the privilege of sending two delegates.'"

The amendment lies over until the next meeting of the Association, under a rule of the organization.

On motion of Dr. Palmer, the resolutions reported at the last annual meeting of the Association by the Committee on Plans of Organization for State and County Medical Societies, were taken up and adopted.

The following resolutions were offered and adopted :

By Dr. Pitcher—

*Resolved,* That the members of this Association, as recipients of the cordial, generous, and elegant hospitalities extended to them by the profession and the citizens of Nashville, in placing on record an expression of thanks for the social amenities they have enjoyed during its tenth annual session, wish also to leave behind them tne assurance, that the recollection of their short sojourn in Tennessee, will be cherished as dearly as the remembrance of the far off sound of water, by the exhausted and way-worn traveler.

By Dr. Means—

*Resolved,* That the earnest thanks of this body be presented to the authorities of the State and city, who have tendered this magnificent State Capitol for their sittings during the present session.

By Dr. Currey—

*Resolved,* That the thanks of the Association be tendered to the reporters of the city press, for the accuracy and promptness with which they have reported the proceedings of the Association, and to the publishers, for the liberal supply of their morning papers during the session of the Association.

By Dr. Wistar—

*Resolved,* That the thanks of this meeting be presented to Dr. Wm. Brodie, for the efficiency with which he has discharged the duties of Secretary.

By Dr. Byford—

*Resolved,* That the State and County Societies throughout the

Union be requested to recommend to their members to purchase the Transactions of the American Medical Association, and that their officers act as agents for the same.

On motion of Dr. Gunn, of Michigan, the Association recognized the presentation of a pamphlet by Henry Frazer Campbell, M. D., claiming "Priority in the discovery and naming of the Excito-Secretory System of Nerves."

On motion of Dr. Byford, the Association adjourned *sine die.*

